# Synthesis, X-Ray Structure, and Characterization of *Catena*-bis(benzoate)bis*{* *N*,*N*-bis(2-hydroxyethyl)glycinate}cadmium(II)

**DOI:** 10.1155/2010/281932

**Published:** 2010-10-10

**Authors:** Eugenia Katsoulakou, Konstantis F. Konidaris, Catherine P. Raptopoulou, Vassilis Psyharis, Evy Manessi-Zoupa, Spyros P. Perlepes

**Affiliations:** ^1^Department of Chemistry, University of Patras, 265 04 Patras, Greece; ^2^Institute of Materials Science, NCSR “Demokritos”, 153 10 Aghia Paraskevi Attikis, Greece

## Abstract

The reaction of *N*, *N*-bis(2-hydroxyethyl)glycine (bicine; bicH_3_) with Cd(O_2_CPh)_2_ · 2H_2_O in MeOH yielded the polymeric compound [Cd_2_(O_2_CPh)_2_(bicH_2_)_2_]_n_(**1**). The complex crystallizes in the tetragonal space group *P*4_1_2_1_2. The lattice constants are *a* = *b* = 12.737(5) and c = 18.288(7) Å. The compound contains chains of repeating {Cd_2_(O_2_CPh)_2_(bicH_2_)_2_} units. One Cd^II^ atom is coordinated by two carboxylate oxygen, four hydroxyl oxygen, and two nitrogen atoms from two symmetry-related 2.21111 (Harris notation) bicH_2_^−^ ligands. The other Cd^II^ atom is coordinated by six carboxylate oxygen atoms, four from two bicH_2_^−^ ligands and two from the monodentate benzoate groups. Each bicinate(-1) ligand chelates the 8-coordinate, square antiprismatic Cd^II^ atom through one carboxylate oxygen, the nitrogen, and both hydroxyl oxygen atoms and bridges the second, six-coordinate trigonal prismatic Cd^II^ center through its carboxylate oxygen atoms. Compound **1** is the first structurally characterized cadmium(II) complex containing any anionic form of bicine as ligand. IR data of **1** are discussed in terms of the coordination modes of the ligands and the known structure.

## 1. Introduction

There are many areas illustrating the importance of cadmium coordination and bioinorganic chemistry and the need for further research in this field. The mobilization and immobilization of Cd^II^ in the environment, in the organisms, and in some technical processes can depend significantly on the complexation by chelating organic ligands [1]. For example, anthropogenic chelators released into the environment, humic acids, and several types of ligands produced by microorganisms contribute to the transfer of this metal ion between solid and aqueous phases [2]. Examples of applied cadmium coordination chemistry are found in wastewater treatment and organic separation problems [1, 3]. Cadmium is also important in the interdisciplinary field of Bioinorganic Chemistry. Though Cd^II^ probably does not have any biological function, the body of a normal human adult usually contains some milligrams of it [4], mainly in metallothioneins, where it is tightly bonded to cysteinyl sulfur atoms [5]. In special cases of cadmium poisoning, the so-called “chelation therapy” can be applied in which synthetic chelators, like EDTA^4−^ and 2,3-dimercapto-1-propanol (BAL), are given as antidotes [6]. A number of research groups have been also using ^113^Cd NMR spectroscopy as a “spin spy” in the study of Zn^II^-containing proteins [7]. Systematic comparative studies on the coordination chemistry of Cd^II^ and Zn^II^ with ligands containing donor groups of biological relevance are useful in this topic. The stereochemical adaptability of this d^10^ metal ion favours structural variations, and this fact makes Cd^II^ a central “player” in the fields of Crystal Engineering and Metallosupramolecular Chemistry [8, 9].

**Scheme 1:**
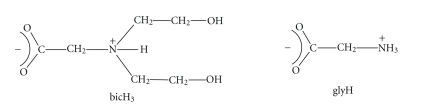
Formulae of *N*,*N*-bis(2-hydroxyethyl)glycine (bicine; bicH_3_) and glycine (glyH) discussed in the paper.

Amongst the ligands that have never been used for the preparation and study of Cd^II^ complexes, neither in the solid state nor in solution, is *N*,*N*-bis(2-hydroxyethyl)glycine, generally known as bicine (bicH_3_, Scheme 1). This is a currently “hot” ligand in Bioinorganic Chemistry. Bicine was first prepared in 1926 by Kiprianov and subsequently became a widely used buffer substance in many biochemical studies [11]. As with its parent compound, the amino acid glycine (glyH) also shown in Scheme 1, the monoanion of bicine, that is, the bicinate (−1) ion (bicH_2_^−^), forms metal complexes. The stability constants of many divalent transition metal complexes of bicinate (−1) have been determined, and it has been found that the [M(bicH_2_)(H_2_O)_x_]^+^ species is always the predominant species in solution [12]. It has repeatedly emphasized [13–15] that as a consequence of its strong complexation properties, the use of bicine as a pH buffer in biochemical or medical studies under the assumption that only little (or no) interaction with divalent metal ions occurs is not justified. It has been shown that not only do bicH_3_ and related compounds buffer H^+^ concentrations but also the resultant metal complexes buffer H^+^ and metal ion concentrations; therefore the employment of bicH_3_ as a buffer requires great care to avoid conflicting data and erroneous conclusions [13–15]. Even though bicinate metal complexes have been studied in solution for years [12–18], mainly through the excellent research of Sigel [12], only few metal complexes have been structurally characterized in the solid state through single-crystal, X-ray crystallography. In those structural studies it was found (see “Results and Discussion”) that the anionic bicH_2_^−^, bicH^2−^, and bic^3−^ ligands are versatile and behave in a variety of terminal and bridging modes. Due to this versatility, the anionic forms of bicine are promising ligands for the isolation of polynuclear transition metal complexes (clusters) [19, 20]. Transition metal cluster chemistry is a currently “hot” research field in contemporary inorganic chemistry [21].

In this paper we report the amalgamation of the above-mentioned two research areas by reporting the preparation, structural characterization, and spectroscopic study of the* first* cadmium(II) bicinate complex. This paper can be considered as a continuation of our interest in the coordination chemistry of bicine [11] and in the Cd^II^ carboxylate chemistry [22].

## 2. Experiments

All manipulations were performed under aerobic conditions using materials and solvents as received. Cd(O_2_CPh)_2_·2H_2_O was prepared by the reaction of Cd(O_2_CMe)_2_·2H_2_O with an excess of PhCO_2_H in CHCl_3_ under reflux. C, H, and N analyses were performed with a Carlo Erba EA 108 analyzer. IR spectra (400–450 cm^−1^) were performed with a Perkin-Elmer PC16 FT-IR spectrometer with samples prepared as KBr pellets.

**[Cd_2_(O_2_CPh)_2_(bicH_2_)_2_]_n_ (1)**

Solid bicH_3_ (0.120 g, 0.74 mmol) was added to a colourless solution of Cd(O_2_CPh)_2_·2H_2_O (0.289 g, 0.74 mmol) in MeOH (40 cm^3^); the solid soon dissolved. The solution was refluxed for 20 min and allowed to slowly evaporate at room temperature. Well-formed, X-ray quality colourless crystals of the product appeared within a period of three days. The crystals were collected by vacuum filtration, washed with cold MeOH (2 × 2 cm^3^) and Et_2_O (3 × 5 cm^3^), and dried in air. The yield was ca. 75%. Found %: C, 39.12; H, 3.97; N, 3.50. Calc % for C_26_H_34_N_2_O_12_Cd_2_: C, 39.46; H, 4.34; N, 3.54. IR data (KBr, cm^−1^): 3235 (sb), 3070 (mb), 2972 (m), 2940 (w), 2894 (w), 1606 (s), 1582 (s),1490 (w), 1445 (w), 1418 (m), 1384 (s), 1334 (m), 1264 (m), 1237 (m), 1174 (w), 1157 (w), 1138 (m), 1069 (s), 1017 (s), 992 (w), 943 (m), 885 (s), 846 (m), 797 (w), 727 (s), 608 (m), 584 (m), 552 (w).

### 2.1. X-ray Crystallography

X-ray data were collected at 298 K using a Crystal LOGIC dual Goniometer diffractometer with graphite-monochromated Mo-K_a_ radiation (*λ* = 0.71073 Å). The appropriate crystal was mounted in air and covered with epoxy glue. Unit cell dimensions were determined and refined by using the angular settings of 25 automatically centered reflections in the range 11 < 2*θ* < 23°. Intensity data were recorded using a **θ**–2**θ**scan. Three standard reflections showed less than 3% variation and no decay. Lorentz polarization and Ψ-scan absorption corrections were applied using Crystal Logic software. The structure was solved by direct methods using SHELXS-97 [23] and refined by full-matrix least-squares techniques on *F* ^2^ with SHELX-97 [24]. Hydrogen atoms were located by difference maps and refined isotropically, except those on O(3), C(6), and C(15) which were introduced at calculated positions as riding on bonded atoms with *U* equal 1.3 times the *U*(eq) of the respective atom. All nonhydrogen atoms were refined anisotropically. CCDC 771321 contains the supplementary crystallographic data for this paper. This data can be obtained free of charge at http://www.ccdc.cam.ac.uk/conts/retrieving.html [or from the Cambridge Crystallographic Data Centre, 12 Union Road, Cambridge CB2 1EZ, UK; Fax: ++44-1223-336 033; E-mail: deposit@ccdc.cam.ac.uk]. Important crystal data and parameters for data collection and refinement are listed in Table 1.

## 3. Results and Discussion

### 3.1. Synthetic Comments

Treatment of bicH_3_ with 1.5 equivalent of Cd(O_2_CPh)_2_·2H_2_O in refluxing MeOH gave a colourless solution from which complex [Cd_2_(O_2_CPh)_2_(bicH_2_)_2_]_n_(1) was obtained in 60% yield (based on the ligand). Its formation can be represented by the stoichiometric equation

(1) $$\begin{array}{c} 2\text{n}\;\text{Cd}{\left({\text{O}}_{2}\text{CPh}\right)}_{2}\cdot 2{\text{H}}_{2}\text{O}+2\text{n}\;{\text{bicH}}_{3}\underset{\text{T}}{\overset{\;\;\;\;\;\;\text{MeOH}\;\;\;\;\;}{\to }}\\ {\left[{\text{Cd}}_{2}{\left({\text{O}}_{2}\text{CPh}\right)}_{2}{\left({\text{bicH}}_{2}\right)}_{2}\right]}_{\text{n}}+2\text{n}\;{\text{PhCO}}_{2}\text{H}+4\text{n}\;{\text{H}}_{2}\text{O} \end{array}$$

The “wrong” Cd^II^ to bicH_3_ reaction ratio (1.5 : 1) employed for the preparation of **1 **did not prove detrimental to the formation of the product. With the identity of **1** established by single-crystal X-ray crystallography, the “correct” stoichiometry (1 : 1) was employed and led to the pure compound in 75% yield (see Section 2).

The PhCO_2_^−^ group present in the reaction mixture plays a double role. It helps the deprotonation of bicH_3 _ and participates in the complex as ligand. 

As a next step we decided to use a large excess of Cd(O_2_CPh)_2_·2H_2_O (Cd^II^ : bicH_3_ = 3 : 1) or to add base (LiOH, Et_3_N, Bu^n^_4_NOH) in the reaction mixture targeting the double or/and triple deprotonation of bicine. We repeatedly isolated a powder, analyzed as Cd_2_(O_2_CPh)(bic)(H_2_O)_2_, but we could not crystallize it; thus this second product has yet to be structurally characterized.

### 3.2. Description of Structure

**Figure 1:**
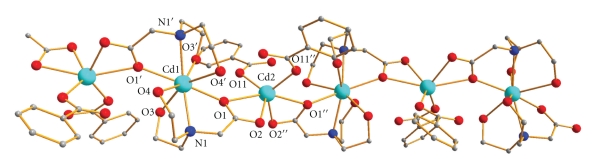
Partially labeled plot of a portion of the chain that is present in complex **1**. Single and double primes are used for symmetry-related atoms (see footnote of Table 2).

Selected interatomic distances and angles for complex **1** are listed in Table 2. The molecular structure of the compound is shown in Figure 1.

**Scheme 2:**
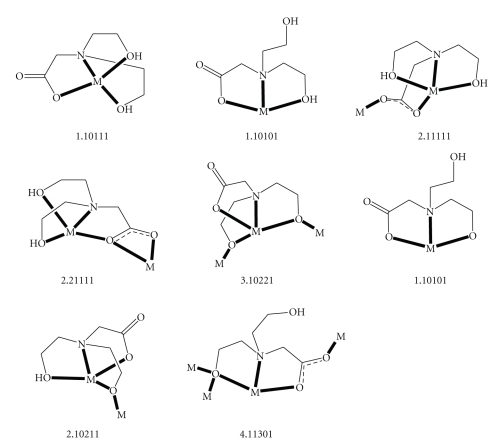
The to-date crystallographically established coordination modes of the bicH_2_^−^, bicH^2−^, and bic^3−^ ligands and the Harris notation in [10] that describes these modes.

The compound contains chains of repeating {Cd_2_(O_2_CPh)_2_(bicH_2_)_2_} units. Each unit contains two crystallographically independent Cd^II^ atoms [Cd(1), Cd(2)] which lie on crystallographic twofold axes. Cd(1) is coordinated by two carboxylate oxygen atoms [O(1), O(1′)], four hydroxyl oxygen atoms [O(3), O(4), O(3′), O(4′)] and two nitrogen atoms [N(1), N(1′)] from two symmetry-related bicinate(−1), that is, bicH_2_^−^, ligands. Cd(2) is coordinated by six carboxylate oxygen atoms; four of them [O(1), O(1′′), O(2), O(2′′)] belong to two symmetry-related bicH_2_^−^ ligands, and two [O(11), O(11′′)] come from two symmetry-related monodentate PhCO_2^−^_ groups. Each bicH_2_^−^ simultaneously chelates Cd(1) through one carboxylate oxygen, the nitrogen, and both hydroxyl oxygen atoms forming three stable, 5-membered chelating rings and bridges Cd(2) through its carboxylate oxygen atoms; thus, one carboxylate oxygen atom [O(1)] of bicH_2_^−^ is *μ*_2_. Adopting Harris notation in [10], the crystallographically unique bicH_2_^−^ group behaves as a 2.21111 ligand (Scheme 2).

The Cd-O_carboxylate_ bond distances are in the wide range 2.190(3)–2.550(2) Å. The bridging Cd-O(1) distances [2.373(2), 2.550(2) Å] are asymmetric. The Cd(2)-O(1) bond distance for the bridging bicinate carboxylate oxygen atom is longer than the distance exhibited by the terminal oxygen atom [O(2)] to the same Cd^II^ atom [2.550(2) versus 2.311(4) Å]. The increase in bond length upon bridging relative to terminal ligation has been observed previously [22] in complexes containing carboxylate ligands with one bridging oxygen atom. Based on theoretical and experimental studies which have indicated that the* syn*-lone pairs of the carboxylate group are more basic than the *anti*-lone pairs [38], one might expect the Cd(2)-O(1) distance to be shorter than the Cd(1)-O(1) distance; however, the reverse relation holds for** 1** (see Table 2). This result, which is in accordance with other Cd^II^ carboxylate complexes [22], suggests that the Cd-O bond lengths involving *η* ^1^ : *η* ^2^: *μ*_2_ carboxylate groups are mainly influenced by geometrical factors rather than the electronic properties of the carboxylate group. The Cd(2)-O bond lengths agree well with values found for other 6-coordinate cadmium(II) carboxylate complexes [39, 40]. The average value for the Cd(2)-O bond distances [2.350(4) Å] is smaller than that for the Cd(1)-O ones [2.435(3) Å], due to the lower coordination of Cd(2) compared to the coordination number of Cd(1) [6 versus 8]. The intrachain Cd(1)⋯Cd(2) distance is 4.739(2) Å.

**Figure 2:**
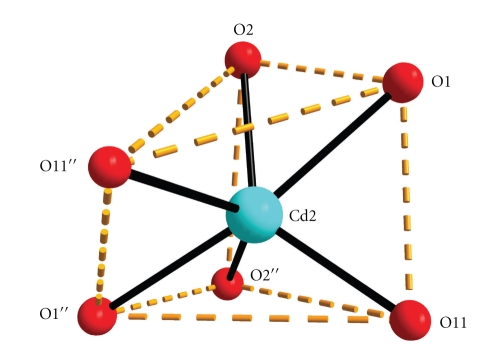
The distorted trigonal prismatic geometry of Cd(2) in complex **1**. Double primes are used for symmetry-related atoms (see footnote of Table 2).

**Figure 3:**
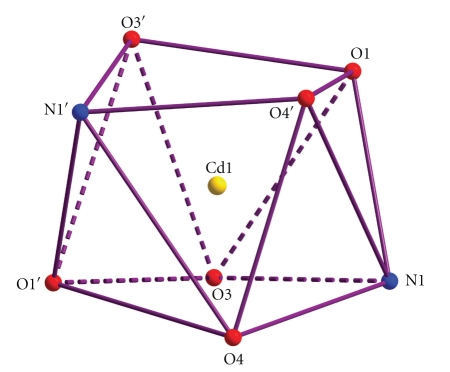
The distorted square antiprismatic stereochemistry about Cd(1) in complex **1**. The coordination bonds have not been drawn for clarity. Primes are used for symmetry-related atoms (see footnote of Table 2).

The coordination geometry of Cd(2) can be described as a very distorted trigonal prismatic (Figure 2). The two carboxylate oxygen atoms of bicH_2_^−^ and the benzoate oxygen atom constitute each trigonal face. The angles of triangular faces are in the wide range 32.9–91.9°. The two trigonal faces are not parallel, with the planes defined by O(1)-O(2)-O(11′′) and O(11′′)-O(2′′)-O(11) making an angle of 27.6°. The coordination polyhedron of the donor atoms about Cd(1) is best described as a distorted square antiprism (Figure 3). Since even the more stable of the possible 8-coordinate geometries (square antiprismatic, triangular dodecahedral, and cubic) differ slightly in energy from one another, the geometry observed may be largely a reflection of constraints placed on the complex by ligand requirements and packing considerations. 

Compound **1** is hydrogen bonded. Metric parameters for the bonds are listed in Table 3. The O-H⋯O hydrogen bonds are intrachain. Both hydroxyl oxygen atoms [O(3), O(4)] are involved as donors, while both the coordinated [O(11)] and uncoordinated [O(12)] benzoate oxygen atoms act as acceptors. A weak interchain hydrogen bond, involving one benzoate carbon atom [C(16)] as donor and the terminally ligated carboxylate oxygen atom [O(2)] of a bicH_2_^−^ ligand from a neighbouring chain as acceptor, is responsible for the formation of a 2D network.

Compound **1** joins a family of mononuclear, polynuclear, and polymeric complexes with the mono- (bicH_2_^−^), di- (bicH^2−^), and trianionic (bic^3-^) derivatives of bicine as ligands [11, 19, 20]. The members of this family are listed in Table 4, together with the coordination modes of the bicinate ligands for convenient comparison. The to-date crystallographically established coordination modes of bicH_2_^−^, bicH^2−^, and bic^3-^ are shown in Scheme 2. Compound **1** is the first cadmium(II) bicinate complex which has been structurally characterized. The bicH_2_^−^ ligand in **1 **adopts the extremely rare coordination mode 2.21111; see Scheme 2. This ligation mode has been observed in the past only in the 1D coordination polymer {Mn_2_(bicH_2_)_2_(H_2_O)_2_]Br_2_·2H_2_O}_n_ [36], in which the Mn^II^ ions are 7 coordinate with a slightly distorted pentagonal bipyramidal coordination geometry.

### 3.3. IR Spectroscopy

IR assignments of selected diagnostic bands for bicH_3 _(the free ligand exists in its zwitterionic form in the solid state with the carboxylic group being deprotonated and the tertiary nitrogen atom protonated [41]) and complex **1** are given in Table 5.

The IR spectrum of complex **1** exhibits a medium intensity, broad band at 3070 cm^−1^, attributable to the O-H stretching vibration of the bicinate(−1) ligand [11, 27, 28]. The broadness and low frequency of this band are both indicative of strong hydrogen bonding [11]. The *ν*(OH)_bicH_2_^−^_ mode is situated at lower frequencies in the spectrum of **1** than for free bicH_3_ (at 3190 and 3090 cm^−1^ [28]); this shift is consistent with the coordination of the –OH groups. The *ν*_as_(CO_2_)_bicH_2_^−^_ and *ν*_s_(CO_2_)_bicH_2_^−^_ bands of **1 **appear at 1582 and 1418 cm^−1^ [11]. The corresponding bands of free, zwitterionic bicH_3_ are at 1639 and 1401 cm^−1^ [28, 29]. The fact that Δ_complex_ (164 cm^−1^) <Δ_bicH_3__ (238 cm^−1^), where Δ = *ν*_as_(CO_2_) − *ν*_s_(CO_2_)_, _ is in accordance with the crystallographically established chelating-bridging mode (*η* ^1^:*η* ^2^:*μ*_2_) of the bicinate(−1) carboxylate group [42]. The strong bands at 1606 and 1384 cm^−1^ in the spectrum of** 1** are assigned to the *ν*_as_(CO_2_) and *ν*_s_(CO_2_) modes of the benzoate ligands, respectively [42]. The parameter Δ is 222 cm^−1^ significantly larger than that for NaO_2_CPh (184 cm^−1^), as expected for the monodentate mode of benzoate ligation [42].

## 4. Conclusions and Perspectives

Complex **1** covers a gap in literature, because it is the first structurally characterized cadmium(II) bicinate compound. The bicinate(−1) ligand adopts the extremely rare pentadentate 2.21111 coordination mode, while the two crystallographically independent Cd^II^ centers are found in two different stereochemistries.

The results presented here support our belief that the bicH_3_/RCO_2_^−^ (R = various) ligand “blends” may be effective generators of interesting structural types in the chemistry of other transition metals. Reactions of CdCl_2_, CdBr_2_, CdI_2_, and Cd(NO_3_)_2_ with bicH_3_ have not been studied to date, and we do believe that the structural types of the products will be dependent on the particular nature of the Cd^II^ source. Analogues of **1** with zinc(II) have not yet been reported, but preliminary results in our laboratories indicate completely different chemistry compared with that of cadmium(II). Synthetic efforts are also in progress to “activate” the potential of bicH^2−^ and bic^3−^ to bridge more than four metal ions.

## Figures and Tables

**Table 1:** Crystal data and structure refinement for complex **1**.

Empirical formula	C_26_H_34_Cd_2_N_2_O_12_
Formula weight	791.35
Crystal size (mm)	0.20 × 0.23 × 0.50
Crystal system	Tetragonal
Space group	*P*4_1_2_1_2
Flack parameter *x *	0.01(3)
**θ** range for data collection (°)	1.95 ≤ *θ* ≤ 25.04
*a*, Å	12.737(5)
*b*, Å	12.737(5)
*c*, Å	18.288(7)
*α*°	90
*β*°	90
*γ*°	90
*V*, Å^3^	2967(2)
*Z*	4
*ρ*_calcd_, g cm^−3^	1.772
*μ*, mm^−1^	1.498
*F*(000)	1584
*Limiting indices*, (°)	0 ≤ *h* ≤ 15
	0 ≤ *k* ≤ 15
	−21 ≤ *l* ≤ 21
Reflections collected	5741
Unique reflections	2632 (*R*_int _ = 0.0454)
Reflections used [*I* > 2*σ*(*I*)]	2448
Parameters	246
*GoF *(on *F * ^2^)	1.047
*R*1^a^	0.0273
*w* *R*2^a^	0.0674
(Δ*ρ*)_max _/(Δ*ρ*)_min _, e Å^−3^	0.742/−0.570

^a^ *I* > 2*σ*(*I*).

**Table 2:** Selected bond lengths (Å) and angles (°) for complex **1** ^a^.

Cd(1)-O(1)	2.373(2)	Cd(2)-O(11)	2.190(3)
Cd(1)-O(3)	2.375(3)	C(1)^b^-O(1)	1.242(5)
Cd(1)-O(4)	2.557(3)	C(1)^b^-O(2)	1.253(5)
Cd(1)-N(1)	2.465(3)	C(11)^c^-O(11)	1.264(5)
Cd(2)-O(1)	2.550(2)	C(11)^c^-O(12)	1.234(5)
Cd(2)-O(2)	2.311(4)		
			
O(1)-Cd(1)-O(1′)	153.4(1)	O(1)-Cd(2)-O(1′′)	156.8(1)
O(1)-Cd(1)-N(1′)	119.8(1)	O(1)-Cd(2)-O(2)	52.9(1)
O(3)-Cd(1)-O(3′)	87.7(2)	O(2)-Cd(2)-O(2′′)	94.2(2)
O(3)-Cd(1)-O(4′)	155.3(1)	O(2)-Cd(2)-O(11)	129.6(1)
O(4)-Cd(1)-O(4′)	75.3(2)	O(11)-Cd(2)-O(11′′)	117.6(2)
O(4)-Cd(1)-N(1′)	84.8(1)	O(11)-Cd(2)-O(2′′)	93.1(1)
N(1)-Cd(1)-N(1′)	147.5(2)	O(1)-C(1)^b^-O(2)	121.2(4)
N(1)-Cd(1)-O(4)	69.3(1)	O(11)-C(11)^c^-O(12)	123.0(4)

^a^Symmetry transformations used to generate equivalent atoms:(′) *y*, *x*, −*z*; (′′) –*y* + 1, −*z* + 1, −*z* + 1/2.

^b^This carbon atom (not labeled in Figure 1) belongs to the carboxylate group of the bicinate(−1) ligand.

^c^This carbon atom (not labeled in Figure 1) belongs to the carboxylate group of the benzoate ligand.

**Table 3:** Dimensions of the hydrogen bonds (distances in Å and angles in °) for complex **1**.

D^a^-H⋯A^b^	D^a^ ⋯ A^b^	H ⋯ A^b^	<D^a^HA^b^	Symmetry code of A
O(3)-H(30)⋯O(11)	2.768(4)	1.91(5)	157(5)	*y*, *x*, − *z*
O(4)-H(40)⋯O(12)	2.730(5)	1.99(4)	168(5)	1 − *x*, 1 − *y*, − 1/2 + *z*
C(16)-H(16)⋯O(2)	3.088(7)	2.38(5)	128(4)	−1/2 + *y*, 1/2 − *x*, − 1/4 + *z*

^a^D = donor atom

^b^A = acceptor atom.

**Table 4:** Formulae and coordination modes of the bicinate(−1, − 2, − 3) groups of the structurally characterized metal complexes containing various forms of bicine as ligands.

Complex^a^	Coordination modes^b^	References
[Cu(bicH_2_)_2_]	1.10101	[25, 26]
[Ni(bicH_2_)_2_]	1.10101	[27]
[CuCl(bicH_2_)]	1.10111	[28]
{[Cu(bicH_2_)](ClO_4_)}_n_	2.11111	[29]
[Cu(SCN)(bicH_2_)]_n_^c^	1.10111	[30]
[CuBr(bicH_2_)]	1.10111	[31]
[CuBr(bicH_2_)(H_2_O)]	1.10111	[31]
[Mn_2_Cl_2_(bicH_2_)_2_]_n_	2.11111	[32]
{[Mn_2_(bicH_2_)_2_(H_2_O)_2_]Br_2_}_n_	2.21111	[33]
[Cu(bicH_2_)(bzimH)](ClO_4_)^d^	1.10111	[34]
[Cu(bicH_2_)(Iq)](ClO_4_)^e^	1.10111	[34]
{[La(bicH_2_)_2_]Cl}_n_	1.10111, 2.11111	[35]
[Gd(O_2_CMe)(bicH_2_)(phen)(H_2_O)](ClO_4_)	1.10111	[11]
[Fe_6_(bic)_6_]	3.10221	[19]
[ReCl(bicH_2_){N = NC(O)Ph}(PPh_3_)]	1.10101	[36]
[ReOCl(bicH)(PPh_3_)]	1.10101	[37]
(Et_2_NH_2_)_2_[Fe_6_O_2_(OH)_2_(O_2_CCMe_3_)_8_(bic)_2_]	3.10221	[20]
[Fe_12_O_4_(O_2_CCMe_3_)_8_(bic)_4_(bicH)_4_]	3.10221^f^, 2.10211^g^, 4.11301^g^	[20]
[Cd_2_(O_2_CPh)_2_(bicH_2_)_2_]_n_	2.21111	This paper

^a^Solvate and other lattice molecules have been omitted.

^b^Using the Harris notation in [10].

^c^The Cu^II^ ions are bridged by the SCN^−^ ligands.

^d^bzimH: benzimidazole.

^e^Iq: isoquinoline.

^f^For the bic^3−^ ligands.

^g^For the bicH^2−^ ligands.

**Table 5:** Most characteristic and diagnostic IR fundamentals (cm^−1^) for bicH_3_ and complex **1**.

Assignment	bicH_3_	**1**
**ν**(OH)	3190 (sb), 3090 (mb)	3070 (mb)
**ν**(CH)	2904 (m), 2844 (w)	2972 (m), 2940 (w), 2894 (w)
*ν*_as_(CO_2_)bicH3/bicH_2_^−^	1644 (sb)	1582 (s)
*ν*_as_(CO_2_)PhCO_2_^−^		1606 (s)
*ν*_s_(CO_2_)bicH3/bicH_2_^−^	1394 (s)	1418 (m)
*ν*_s_(CO_2_)PhCO_2_^−^		1384 (s)
